# Genome-Wide CRISPR-Cas9 Screen Identifies MicroRNAs That Regulate Myeloid Leukemia Cell Growth

**DOI:** 10.1371/journal.pone.0153689

**Published:** 2016-04-15

**Authors:** Jared Wallace, Ruozhen Hu, Timothy L. Mosbruger, Timothy J. Dahlem, W. Zac Stephens, Dinesh S. Rao, June L. Round, Ryan M. O’Connell

**Affiliations:** 1 Department of Pathology, University of Utah, Salt Lake City, Utah, United States of America; 2 Mutation Generation and Detection Core, HSC Core Research Facilities, University of Utah, Salt Lake City, Utah, United States of America; 3 Huntsman Cancer Institute, University of Utah, Salt Lake City, Utah, United States of America; 4 Department of Pathology, UCLA, Los Angeles, California, United States of America; Cincinnati Children's Hospital Medical Center, UNITED STATES

## Abstract

Mammalian microRNA expression is dysregulated in human cancer. However, the functional relevance of many microRNAs in the context of tumor biology remains unclear. Using CRISPR-Cas9 technology, we performed a global loss-of-function screen to simultaneously test the functions of individual microRNAs and protein-coding genes during the growth of a myeloid leukemia cell line. This approach identified evolutionarily conserved human microRNAs that suppress or promote cell growth, revealing that microRNAs are extensively integrated into the molecular networks that control tumor cell physiology. miR-155 was identified as a top microRNA candidate promoting cellular fitness, which we confirmed with two distinct miR-155-targeting CRISPR-Cas9 lentiviral constructs. Further, we performed anti-correlation functional profiling to predict relevant microRNA-tumor suppressor gene or microRNA-oncogene interactions in these cells. This analysis identified miR-150 targeting of p53, a connection that was experimentally validated. Taken together, our study describes a powerful genetic approach by which the function of individual microRNAs can be assessed on a global level, and its use will rapidly advance our understanding of how microRNAs contribute to human disease.

## Introduction

Acute Myeloid Leukemia (AML) is an aggressive hematologic malignancy that carries a poor prognosis. In AML, hematopoiesis is disrupted by the overproduction of transformed myeloid cells, leading to life-threating anemia, immunosuppression, and bleeding due to decreased normal blood cell production. A variety of genetic and epigenetic aberrations are thought to drive leukemic phenotypes, including alterations in protein-coding genes and microRNAs.

MicroRNAs (miRNAs) are small non-coding RNAs that repress their target genes by binding to cognate 3’ UTR sites in their respective mRNA targets, preventing their translation and/or triggering mRNA degradation. miRNA expression is highly dysregulated in AML [1, 2], and certain miRNAs have been shown to modulate leukemia cell biology *in vitro* [3]. Furthermore, the overexpression of a few specific miRNAs is sufficient to induce leukemic transformation in mice [4, 5], whereas other miRNAs act as tumor suppressors via repression of known protein oncogenes in hematopoietic malignancy [6, 7]. However, while the dysregulation of a number of miRNAs has been implicated in leukemia, the functional impact of many miRNAs and their putative targets on leukemic phenotypes remains unclear.

In this study, we took an unbiased, global loss-of-function approach to determine which miRNAs, and which of their putative targets, are involved in MV4-11 cell line growth, a model of myeloid leukemia. Because of the many caveats associated with previously described methods of miRNA loss-of-function screening that limits their use, we employed CRISPR-Cas9 technology [8–10]. Using this approach, each human miRNA and protein-coding gene in MV4-11 cells was individually disrupted and the impact on cellular growth was determined. Results point to a subset of evolutionarily conserved miRNAs that regulate cellular growth, and have also determined the impact of predicted miRNA targets that mediate these effects on tumor cell proliferation and survival. Furthermore, we have validated miR-150 as a critical promoter of leukemic cell growth in our system through targeting of p53. Taken together, our study demonstrates that CRISPR-Cas9 technology can be used to identify novel, functionally relevant miRNAs in mammalian cell phenotypes, while simultaneously identifying putative target proteins with opposing function. Our dataset also provides a resource describing the effects of individual miRNAs and protein-coding genes on leukemic cell fitness.

## Results

### CRISPR-Cas9 screen identifies protein-coding genes that regulate AML cell line growth

**Fig 1:**
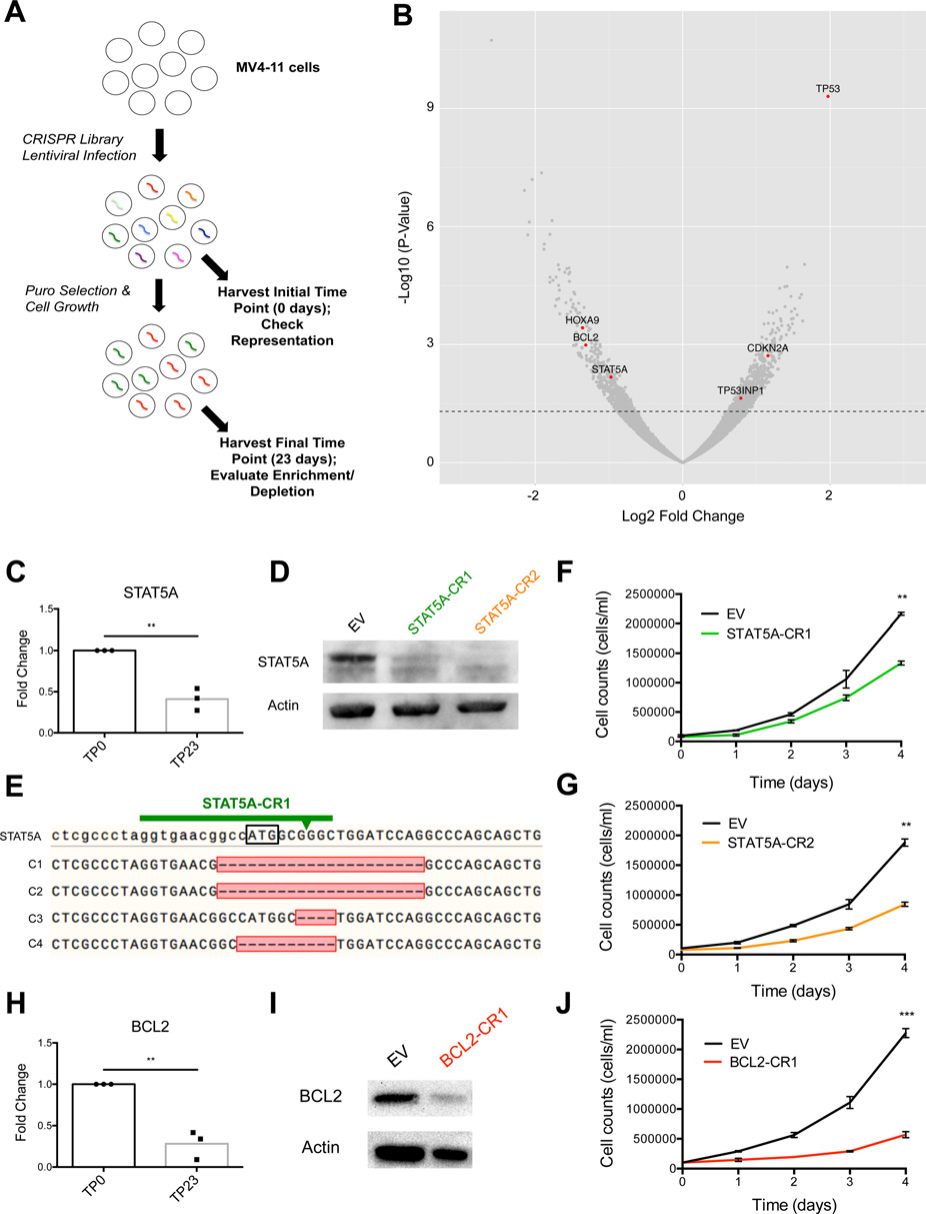
CRISPR-Cas9 loss-of-function screen identifies protein-coding genes, including known oncogenes STAT5A and BCL2, as important for MV4-11 cell line growth. (A) Overall experimental design of lentiCRISPRv2 library screen. (B) Log2 Fold Change of each protein-coding gene targeted in lentiCRISPRv2 library screen (x-axis) plotted against–Log10 P-Value (y-axis). Dotted line represents p-value = 0.05. (C) Fold change of STAT5A normalized read counts in lentiCRISPRv2 library screen as compared to TP0. (D) Western blot of STAT5A using cellular extract from STAT5A-CR1, STAT5A-C2, or EV control infected MV4-11 cells with actin serving as load control. (E) DNA sequencing of the STAT5A locus from four representative STAT5A-CR1 infected MV4-11 clones (C1-C4). STAT5A represents the wild type (WT) sequence. Black box indicates translational start site. Arrow represents predicted cleavage site of Cas9 endonuclease. Red box identifies mutated region, with dashed lines indicating deleted nucleotides. (F, G) Growth curve for STAT5A-CR1 or STAT5A-CR2 infected MV4-11 cells compared to EV control. (H) Fold change of BCL2 normalized read counts in lentiCRISPRv2 library screen as compared to TP0. (I) Western blot of BCL2 in BCL2-CR1 or EV control infected MV4-11 cells with actin serving as load control. (J) Growth curve for BCL2-CR1 infected MV4-11 cells compared to EV control. (B, C, H) Represents combined data from three independently performed lentiCRISPRv2 library infections. Data represented as mean +/- SEM. P-values as indicated: *≤0.05, **≤0.01, ***≤0.001, and ns p>0.05. See also S1 Table.

In order to determine which protein-coding genes and miRNAs regulate leukemic cell growth, we utilized a genome-scale CRISPR-Cas9 library (lentiCRISPRv2 library) [11, 12] to disrupt specific genes and evaluate the impact on cellular fitness over time. The lentiCRISPRv2 library contained 3 unique single guide RNAs (sgRNAs) targeting each protein-coding gene, as well as 4 unique sgRNAs targeting each miRNA gene locus cloned into an all-in-one CRISPR-Cas9 construct (lentiCRISPRv2). MV4-11 cells, a human-derived AML cell line homozygous for the FLT3-ITD mutation [13] and positive for the fusion protein MLL-AF4 [14], were transduced with the lentiCRISPRv2 library at ~250X coverage and an MOI of 0.3 to favor single viral integrations. An initial time point (TP0) was taken two days post-infection to assess library representation. Cells were selected with puromycin (puro) for 7 days, at which point puro was removed and growth was allowed to continue for an additional 16 days before a final time point (TP23) was collected (Fig 1A). Following genomic DNA (gDNA) extraction from cells at both time points and PCR amplification of each sgRNA sequence, we performed Illumina sequencing to generate read counts for each gene-targeting lentiCRISPRv2 construct. In order to accurately determine the impact of each gene on cell fitness over the 23-day time course, we combined the normalized read counts of all lentiCRISPRv2 constructs targeting a given gene at TP23 and expressed this as log2 fold change relative to the initial abundance of constructs at TP0 using DEseq2. We calculated the average log2 fold change across three independently-performed experiments to determine whether loss-of-function of each gene expressed in MV4-11 cells led to increased, decreased, or no change in cell growth over time using a cutoff p-value of 0.05. Furthermore, MV4-11 cells were transcriptionally profiled using RNA sequencing, and only expressed genes were included in our analysis. Using this approach, we identified protein-coding genes whose deletion significantly affected MV4-11 cell growth (Fig 1B and S1 Table). Of the 19,052 protein-coding genes targeted in our screen, we found 715 genes whose deletion consistently resulted in increased cell numbers, which included many known tumor suppressor genes (TSGs). We also identified 516 genes whose deletion reproducibly resulted in decreased cellular growth, including a number of known oncogenes.

Signal transducer and activator of transcription 5A (STAT5A) was among the known oncogenes that our screen identified as important for cell growth (Fig 1B and 1C). STAT5 is a key signaling pathway that is inappropriately activated by FLT3-ITD mutations [15] and promotes FLT3-ITD driven growth. Thus, we tested the efficacy of two distinct STAT5A targeting lentiCRISPRv2 constructs (STAT5A-CR1 and STAT5A-CR2) through individual transduction of MV4-11 cells, and observed significantly reduced STAT5A protein levels by western blotting two weeks post-infection (Fig 1D). We sequenced the STAT5A locus in individual clones transduced with the STAT5A-CR1 vector and found that 80% of cells contained mutations at the expected Cas9 cut site (Fig 1E**).** STAT5A-CR1 and STAT5A-CR2 cells grew at a slower rate than cells transduced with a lentiCRISPRv2 empty vector (EV) control, confirming results from our screen indicating that STAT5A is a promoter of FLT3-ITD+ leukemic cell growth (Fig 1F and 1G). Similar results were obtained when we independently validated another known oncogene, BCL2 (Fig 1H–1J).

**Fig 2:**
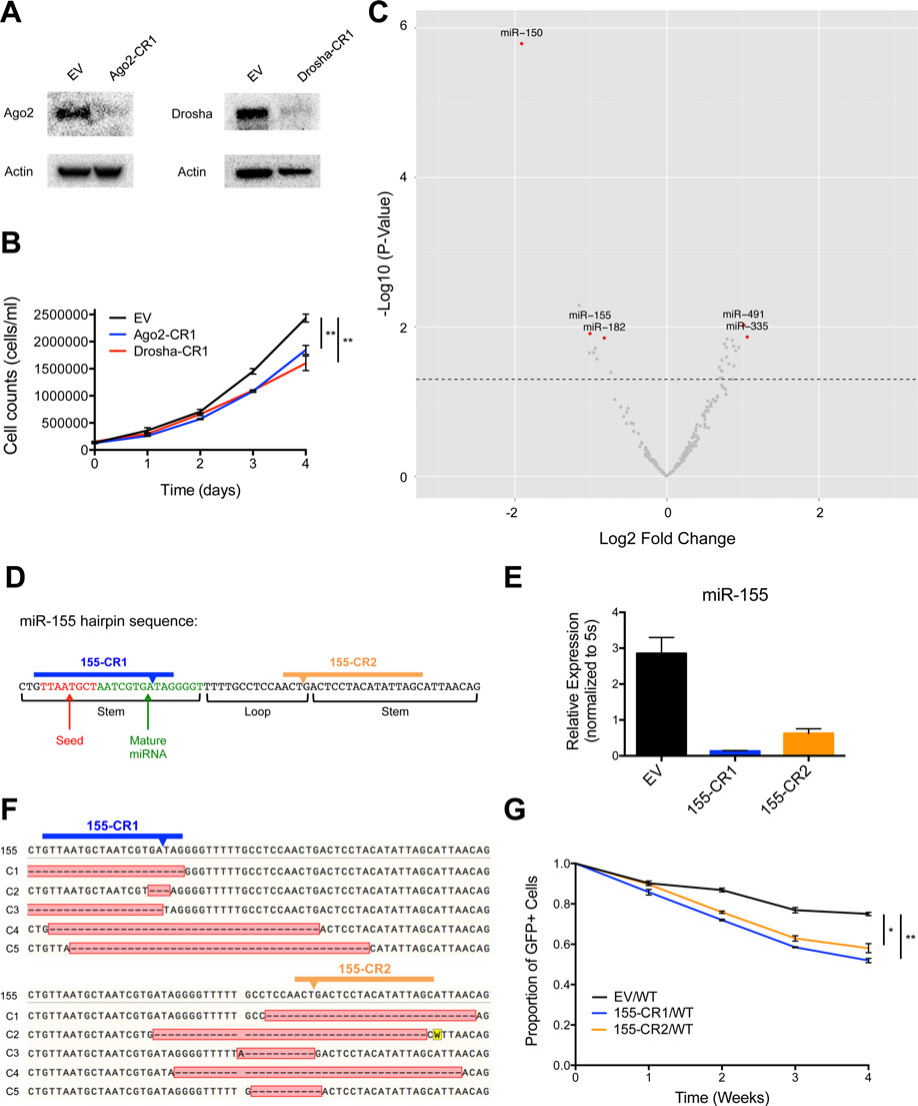
Identification of individual microRNAs, including miR-155, that regulate MV4-11 cell line growth. (A) Western blots of Ago2 and Drosha using cellular extract from Ago2-CR1, Drosha-CR1, and EV infected MV4-11 cell lines with actin serving as load control. (B) Growth curve for Ago2-CR1 and Drosha-CR1 infected MV4-11 cells compared to EV control. (C) Log2 Fold Change of each conserved microRNA gene targeted in lentiCRISPRv2 library screen (x-axis) plotted against–Log10 P-Value (y-axis). Dotted line represents p-value = 0.05. Represents combined data from three independently performed lentiCRISPRv2 library infections. (D) Schematic of miR-155 hairpin sequence as annotated in miRBase and sgRNA design of two independent miR-155-targeting lentiCRISPRv2 constructs (155-CR1, 155-CR2). (E) Expression levels of miR-155 in MV4-11 cells infected with EV control, 155-CR1, or 155-CR2 lentiCRISPRv2 constructs determined by qPCR. Expression normalized to 5s. (F) DNA sequencing of five representative 155-CR1 and 155-CR2 infected MV4-11 clones (C1-C5). 155 represents the WT sequence. Arrow indicates predicted cleavage site of Cas9. Red box identifies the mutated region, with dashed lines indicating deleted nucleotides. (G) Competitive growth curve of EV (GFP+), 155-CR1 (GFP+), or 155-CR2 (GFP+) infected MV4-11 cells mixed ~1:1 with WT MV4-11 cells at time point 0. Y-axis = (%GFP+ cells at indicated time point)/(%GFP+ cells initial). Data represented as mean +/- SEM. P-values as indicated: *≤0.05, **≤0.01, ***≤0.001, and ns p>0.05. See also S2 Table.

Interestingly, we also found that cells with CRISPR-Cas9-mediated depletion of Argonaute 2 (Ago2), Dicer, or Drosha, important proteins in the miRNA processing pathway, displayed reduced cell numbers over time, with p-values that trended towards, but did not reach, statistical significance (Data not shown). Because these genes are all in the miRNA biogenesis pathway, we sought to investigate this observation further. Therefore, we created Ago2 (Ago2-CR1) and Drosha (Drosha-CR1) deleted MV4-11 cell lines by using the lentiCRISPRv2 system to deliver sgRNAs against each of these protein-coding genes, and deletion was confirmed via western blotting (Fig 2A). These cell lines also demonstrated decreased cellular growth compared to EV-infected control MV4-11 cells (Fig 2B), suggesting that microRNAs were playing a net role in promoting cell growth in this context.

### Critical role for specific, evolutionarily conserved miRNAs during MV4-11 cell growth

We next evaluated which individual miRNAs were playing a functional role in regulating MV4-11 cell growth. We found that 27 of the 197 evolutionarily conserved miRNAs targeted in our screen had a positive or negative influence on cell numbers over time with an average fold change p-value of less than 0.05 when the 3 biological replicate experiments were combined (Fig 2C and S2 Table). Only mature miRNAs expressed in MV4-11 cells, as assessed by RNA sequencing, were considered in our analysis. Interestingly, we found that fewer miRNAs had a functional effect during our screen than protein-coding genes. This observation could be explained by the fact that there are fewer miRNA vs protein-coding genes overall. Furthermore, this finding may also be due to the observation that miRNAs typically display only partial repression of their mRNA targets leading to modest changes in protein levels, whereas many protein-coding genes encode essential proteins that regulate core cellular processes required for growth and viability. miR-150, miR-155, and miR-182 were included in our top hits among conserved miRNAs that promote cell growth. Of relevance, all three have previously been implicated in hematopoietic malignancy [16–18]. We also identified conserved miRNAs that acted to repress cell growth in our screen, including miR-491 and miR-335.

miR-155 was identified as a top miRNA candidate that promoted FLT3-ITD+ cell growth (Fig 2C). Interestingly, miR-155 is also the most highly dysregulated miRNA in primary FLT3-ITD+ AML cells compared to FLT3-WT AML or normal CD34+ hematopoietic stem and progenitor cells [1, 2], and has been implicated in regulating the survival and growth of FLT3-ITD+ cells [19]. To independently validate miR-155, we used two distinct lentiCRISPRv2 constructs represented in our library to generate miR-155 deficient FLT3-ITD+ cell lines; one targeting the mature miRNA region of the miR-155 hairpin sequence (155-CR1), and the other targeting the loop (155-CR2) (Fig 2D). We found that cell lines carrying the 155-CR1 or 155-CR2 constructs had significantly decreased levels of mature miR-155 (Fig 2E). We further analyzed the mutations being created by 155-CR1 and 155-CR2, and found that 15/16 clones analyzed (8/8 of 155-CR1; 7/8 of 155-CR2) contained mutations at the predicted Cas9 cut site (Fig 2F). Of these 15 mutations, we observed 12 deletions and 3 insertions, indicating that NHEJ-mediated deletions were favored in these cells. 5 of the 7 clones analyzed that had been transduced with 155-CR1 contained mutations spanning the seed sequence, the critical portion of the mature miRNA that leads to target repression via complementary binding to the 3’ UTR. In the case of 155-CR2, we conclude that deletion of the loop region leads to disrupted biogenesis of mature miR-155. Both 155-CR1 and 155-CR2 cells exhibited decreased competitive cell growth compared to EV control cells (Fig 2G), thus confirming our library findings.

### Anti-correlation functional profiling identifies relevant microRNA-target pairs, including miR-150 and p53

**Fig 3:**
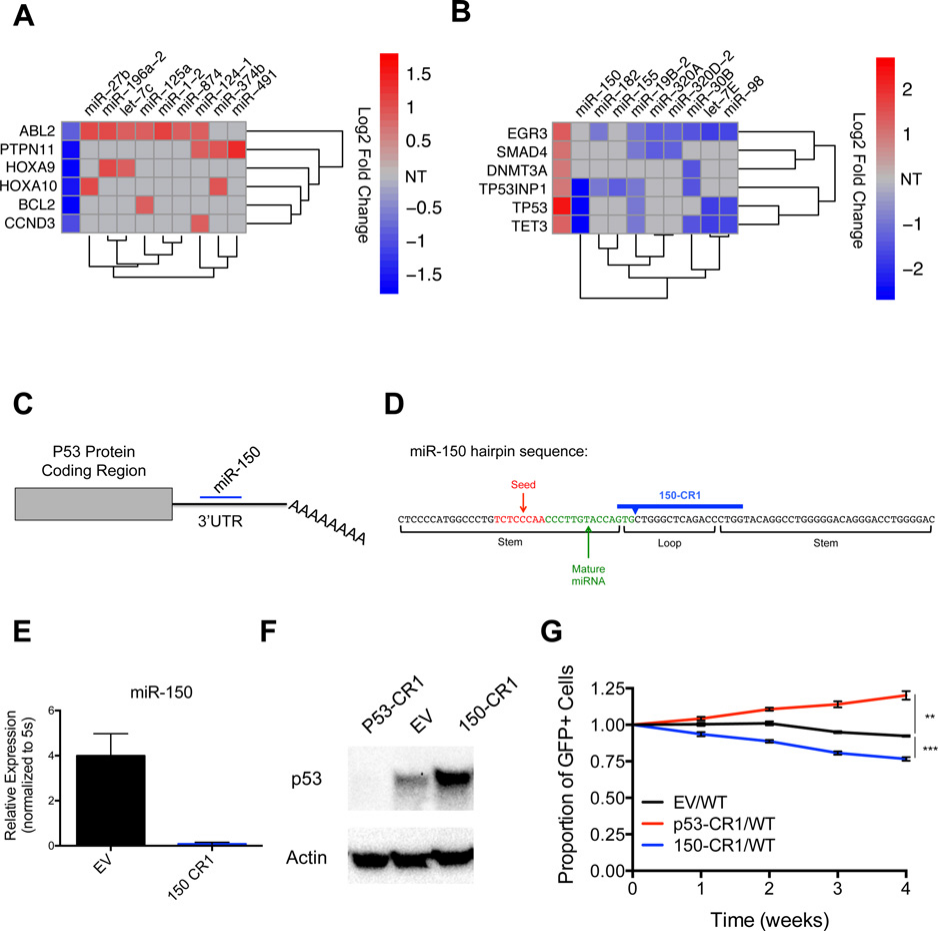
Anti-correlation functional profiling identifies relevant miRNA-target interactions, including miR-150 repression of p53, that regulate MV4-11 cell line growth. (A) Heat map indicating representative oncogenes whose loss leads to decreased cell growth according to Log2 Fold Change values from lentiCRISPRv2 library screen (first column), and functionally anti-correlated miRNAs that are predicted to target each oncogene. Grey boxes indicate that the miRNA is not predicted to bind the 3’UTR of the oncogene (NT = Not targeted). (B) Heat map indicating representative TSGs whose loss lead to increased cell growth according to our Log2 Fold Change values from lentiCRISPRv2 library screen (first column), and miRNAs predicted to target each TSG whose growth anti-correlated in library. Grey boxes indicates that the miRNA is not predicted to bind the 3’UTR of TSG (NT = Not targeted). (C) Schematic showing miR-150 targeting of the p53 3’UTR. (D) Schematic of the miR-150 hairpin sequence as annotated in miRBase and sgRNA design of the miR-150-targeting lentiCRISPRv2 construct (150-CR1). (E) Expression level of miR-150 in MV4-11 cells infected with EV control or 150-CR1 lentiCRISPRv2 constructs determined by qPCR. Expression normalized to 5s. (F) Western blot of p53 in p53-CR1, 150-CR1, and EV control infected MV4-11 cell lines with actin serving as load control. (G) Competitive growth curve of EV (GFP+), p53-CR1 (GFP+), or 150-CR1 (GFP+) infected MV4-11 cells mixed ~1:1 with WT MV4-11 cells at time point 0. Y-axis = (%GFP+ cells at indicated time point)/(%GFP+ cells initial). (A, B) Only expressed protein-coding genes and microRNAs with p-values <0.05 were analyzed. Data represented as mean +/- SEM. P-values as indicated: *≤0.05, **≤0.01, ***≤0.001, and ns p>0.05. See also S3 Table.

We also used our dataset to identify miRNAs predicted to target known oncogenes and TSGs (S3 Table). In the case of several representative oncogenes, their promotion of cell growth inversely correlated with the impact of specific miRNAs with conserved binding sites in their 3’ UTRs (Fig 3A). Similar observations were made for a subset of known TSGs, where their negative effects on cell growth inversely correlated with the impact of specific miRNAs with conserved binding sites in their 3’ UTRs (Fig 3B). This approach, which we refer to as anti-correlation functional profiling, is a powerful method that can be used to globally identify miRNA-target gene pairs that may be functionally linked.

To test the ability of anti-correlation functional profiling to identify functionally relevant miRNA-target pairs in our system, we tested the top association from our miRNA-TSG plot, TP53 (p53) and miR-150. miR-150, the top miRNA hit in our lentiCRISPRv2 library screen, has a conserved binding sequence in the 3’UTR of p53 (Fig 3C), and has been shown to be directly targeted by miR-150 in luciferase reporter assays [20, 21]. We generated a lentiCRISPRv2 construct designed to cut near the mature miRNA coding sequence using one of the miR-150 sgRNAs (150-CR1) represented in the lentiCRISPRv2 library (Fig 3D), and confirmed that 150-CR1 containing MV4-11 cells displayed a significant decrease in miR-150 levels compared to EV control cells by qPCR (Fig 3E). We also generated p53 deficient cell lines (p53-CR1), again using a sgRNA from the lentiCRISPRv2 library cloned into a lentiCRISPRv2 construct. We confirmed that p53 protein levels were reduced in the p53-CR1 cells compared to EV control cells (Fig 3F), and saw an increase in p53 protein level in the 150-CR1 cells compared to EV control, indicating that miR-150 is indeed repressing p53 in MV4-11 cells. Finally, we observed that p53-CR1 cells had a competitive growth advantage compared to EV infected cells (Fig 3G), while 150-CR1 cells had a growth disadvantage. These results validate our anti-correlation functional profiling approach to finding relevant miRNA-target pairs that regulate specific cellular phenotypes, and point to miR-150 repression of p53 as a significant pro-growth and survival mechanism in at least some types of myeloid leukemias.

## Discussion

There are over 1000 different miRNAs in human cells, each with the potential to be functionally relevant in diseases such as cancer. However, the use of large-scale loss-of-function screening to identify functionally relevant miRNAs has been hampered by technical limitations, including the inability of shRNAs to effectively block miRNA biogenesis and function. Most miRNAs that have been studied to date have been assessed on an individual basis. While this has provided important insights into the roles of those examined, many miRNAs have been left uncharacterized. Consequently, there is a tremendous need for high throughput approaches to identifying functionally relevant miRNAs in an unbiased manner to obtain a comprehensive list of miRNAs that impact specific phenotypes. Here, we have successfully used CRISPR-Cas9 technology to carry out a miRNA loss-of-function screen, and although further validation of individual hits is ultimately necessary in more physiologically relevant systems, our results clearly demonstrate that subsets of specific miRNAs act to positively or negatively control cellular proliferation and survival in the system under study and provide a resource to guide future work.

Because miRNAs repress protein-coding target genes, data from our screen could be used to predict functionally relevant miRNA-target gene interactions that regulate leukemic cell growth. Using this anti-correlation functional profiling approach, we identified miRNAs with functions that oppose a specific TSG or oncogene predicted to be a conserved target of each respective miRNA. Further, we confirmed that miR-150 repression of p53 is a promoter of cell growth in our system, which validates this approach. Although additional miRNA-target connections in this setting require further validation, our results underscore the potential of this approach to identify novel miRNA-target networks with relevance to cancer, and do so during a single experiment.

Our screen also identified miR-155, a miRNA that has been clinically connected to FLT3-ITD+ AML, as a promoter of FLT3-ITD+ cell proliferation. To validate this result, we confirmed that two independent sgRNAs against the human miR-155 hairpin sequence were able to dramatically reduce production of mature miR-155. While one of these sgRNAs targeted mature miR-155, including the seed sequence, the other targeted the loop region of the hairpin. These results indicate that, although miRNA hairpin sequences are short, one can find multiple CRISPR-Cas9 sites that can be used to disrupt miRNA biogenesis and subsequently validate and study loss-of-function phenotypes.

Beyond regulating tumor cell proliferation and survival, miRNAs have been implicated in other aspects of cancer, including drug resistance and metastasis [22, 23]. Our current approach has the potential to be used to identify specific miRNAs and their targets that regulate these deleterious processes, and reveal key miRNA species that represent promising therapeutic targets in these contexts. Together, these approaches will provide a systematic view of the molecular networks that coordinate malignant disease origin and subsequent outcomes with a focus on functional relevance.

## Material and Methods

### Cells and tissue culture

MV4-11 cells were purchased from ATCC and used for all *in vitro* experimentation. Cells were cultured in RPMI based media supplemented with 10% FBS, and kept at 37°C with 5% CO_2_. Cells were passaged every 2–3 days in order to stay within 1x10^5^-1x10^6^ cells/ml to maintain logarithmic growth.

### CRISPR-Cas9 library screen and individual LentiCRISPRv2 infections

Genome-scale CRISPR Knock-Out (GeCKO) v2.0 was purchased from Addgene for application in all lentiCRISPRv2 library screens, and performed as described in S1 Supporting Methods. In brief, cells were infected, selected with puromycin, DNA was extracted, the integrated sgRNAs were then amplified and amplicons were subjected to DNA-Seq. Single CRISPR-Cas9 vector infections were performed using a similar approach. Unique sgRNA sequences were cloned into a lentiCRISPRv2 construct (a gift from Feng Zhang; Addgene plasmid #52961) containing either a puro resistance or GFP selection marker. Sequences can be found in our supplemental methods section. The CRISPR-Cas9 library screen data have been deposited in NCBI’s Gene Expression Omnibus under GEO: GSE71544.

### Growth curves and competition assays

Growth curves using STAT5A-CR1, STAT5A-CR2, Drosha-CR1, or Ago2-CR1 cells were performed in 2 ml triplicate cultures in a 6 well plate. EV infected cells were grown in parallel as a positive control. Cells were split to 100,000 cells/ml and counted daily via microscopy using a hemacytometer and trypan blue exclusion until cells reached ~2x10^6^ cells/ml, or the point when cells no longer demonstrated logarithmic growth. Competitive growth assays were performed by mixing miR-155-CR1, miR-155-CR2, p53-CR1, miR-150-CR1, or EV control cells (all GFP+) at a 1:1 ratio with WT MV4-11 cells (GFP-), and measuring the percentage of GFP+ cells over a 4-week time course via flow cytometry.

### Quantitative PCR

Total RNA was isolated from MV4-11 cell lines and mouse BM cells using the miRNeasy spin column kit (Qiagen). Mature miR-155 or miR-150 was quantified using the miRCURY LNA Universal RT microRNA PCR cDNA Synthesis Kit II (Exiqon) and ExiLENT SYBR Green master mix kit (Exiqon) on a Light Cycler 480 PCR machine (Roche). Human or mouse miR-155 LNA primers, human miR-150 LNA primers, and 5S rRNA loading control primers were purchased from Exiqon.

### Expression profiling

Total RNA was isolated using the miRNeasy spin column kit (Qiagen). Expression of small RNAs and long RNAs in MV4-11 cells was performed using RNA sequencing as described further in S1 Supporting Methods. Data have been deposited into GEO as GSE71544.

### Western blot analysis

Total protein extracts from MV4-11 cell lines were harvested using RIPA lysis buffer with protease inhibitors, and protein concentration was determined using a Bio-Rad Protein Assay Dye Reagent kit. SDS-denatured protein was separated via gel electrophoresis and transferred onto a nitrocellulose membrane. Protein was detected via overnight antibody staining with the following antibodies: STAT5A (Santa Cruz L-20), Drosha (Cell Signaling D28B1), Ago2 (Cell Signaling C34C6), p53 (Santa Cruz FL-393), and Actin (Sigma A5441).

### Statistics

Significant p-values were determined using an unpaired Student’s t-test, unless otherwise noted. P-values for lentiCRISPRv2 library screen were determined using DEseq2. Quantitative data are displayed as mean +/- SEM. P-values are shown as indicated: *≤0.05, **≤0.01, ***≤0.001, and ns p>0.05. All statistics were performed in either GraphPad Prizm6.0 or Microsoft Excel. For calculation of p-values in growth curves, individual t-tests were performed for the final time point.

## Supporting Information

S1 Supporting Methods Supplemental experimental procedures. (DOCX)

S1 Table Protein-coding genes significantly affecting MV4-11 cell growth. (XLSX)

S2 Table MicroRNAs significantly affecting MV4-11 cell growth. (XLSX)

S3 Table Anti-correlation functional profiling identifies miRNA-protein coding gene (PCG) pairs that regulate MV4-11 cell growth. (XLSX)

## References

[1] Garzon R, Garofalo M, Martelli MP, Briesewitz R, Wang L, Fernandez-Cymering C, et al Distinctive microRNA signature of acute myeloid leukemia bearing cytoplasmic mutated nucleophosmin. Proceedings of the National Academy of Sciences of the United States of America. 2008;105(10):3945–50. 10.1073/pnas.0800135105 18308931 PMC2268779

[2] Whitman SP, Maharry K, Radmacher MD, Becker H, Mrozek K, Margeson D, et al FLT3 internal tandem duplication associates with adverse outcome and gene- and microRNA-expression signatures in patients 60 years of age or older with primary cytogenetically normal acute myeloid leukemia: a Cancer and Leukemia Group B study. Blood. 2010;116(18):3622–6. 10.1182/blood-2010-05-283648 20656931 PMC2981481

[3] Marcucci G, Mrozek K, Radmacher MD, Garzon R, Bloomfield CD. The prognostic and functional role of microRNAs in acute myeloid leukemia. Blood. 2011;117(4):1121–9. 10.1182/blood-2010-09-191312 21045193 PMC3056468

[4] O'Connell RM, Chaudhuri AA, Rao DS, Gibson WS, Balazs AB, Baltimore D. MicroRNAs enriched in hematopoietic stem cells differentially regulate long-term hematopoietic output. Proceedings of the National Academy of Sciences of the United States of America. 2010;107(32):14235–40. 10.1073/pnas.1009798107 20660734 PMC2922591

[5] Han YC, Park CY, Bhagat G, Zhang J, Wang Y, Fan JB, et al microRNA-29a induces aberrant self-renewal capacity in hematopoietic progenitors, biased myeloid development, and acute myeloid leukemia. The Journal of experimental medicine. 2010;207(3):475–89. 10.1084/jem.20090831 20212066 PMC2839143

[6] Starczynowski DT, Kuchenbauer F, Argiropoulos B, Sung S, Morin R, Muranyi A, et al Identification of miR-145 and miR-146a as mediators of the 5q- syndrome phenotype. Nature medicine. 2010;16(1):49–58. 10.1038/nm.2054 . 19898489

[7] Calin GA, Dumitru CD, Shimizu M, Bichi R, Zupo S, Noch E, et al Frequent deletions and down-regulation of micro- RNA genes miR15 and miR16 at 13q14 in chronic lymphocytic leukemia. Proceedings of the National Academy of Sciences of the United States of America. 2002;99(24):15524–9. 10.1073/pnas.242606799 12434020 PMC137750

[8] Doudna JA, Charpentier E. Genome editing. The new frontier of genome engineering with CRISPR-Cas9. Science. 2014;346(6213):1258096 10.1126/science.1258096 . 25430774

[9] Mali P, Yang L, Esvelt KM, Aach J, Guell M, DiCarlo JE, et al RNA-guided human genome engineering via Cas9. Science. 2013;339(6121):823–6. 10.1126/science.1232033 23287722 PMC3712628

[10] Ran FA, Hsu PD, Wright J, Agarwala V, Scott DA, Zhang F. Genome engineering using the CRISPR-Cas9 system. Nature protocols. 2013;8(11):2281–308. 10.1038/nprot.2013.143 24157548 PMC3969860

[11] Shalem O, Sanjana NE, Hartenian E, Shi X, Scott DA, Mikkelsen TS, et al Genome-scale CRISPR-Cas9 knockout screening in human cells. Science. 2014;343(6166):84–7. 10.1126/science.1247005 24336571 PMC4089965

[12] Sanjana NE, Shalem O, Zhang F. Improved vectors and genome-wide libraries for CRISPR screening. Nature methods. 2014;11(8):783–4. 10.1038/nmeth.3047 . 25075903 PMC4486245

[13] Quentmeier H, Reinhardt J, Zaborski M, Drexler HG. FLT3 mutations in acute myeloid leukemia cell lines. Leukemia. 2003;17(1):120–4. 10.1038/sj.leu.2402740 . 12529668

[14] Andersson A, Eden P, Lindgren D, Nilsson J, Lassen C, Heldrup J, et al Gene expression profiling of leukemic cell lines reveals conserved molecular signatures among subtypes with specific genetic aberrations. Leukemia. 2005;19(6):1042–50. 10.1038/sj.leu.2403749 . 15843827

[15] Mizuki M, Fenski R, Halfter H, Matsumura I, Schmidt R, Muller C, et al Flt3 mutations from patients with acute myeloid leukemia induce transformation of 32D cells mediated by the Ras and STAT5 pathways. Blood. 2000;96(12):3907–14. . 11090077

[16] O'Connell RM, Rao DS, Chaudhuri AA, Boldin MP, Taganov KD, Nicoll J, et al Sustained expression of microRNA-155 in hematopoietic stem cells causes a myeloproliferative disorder. The Journal of experimental medicine. 2008;205(3):585–94. 10.1084/jem.20072108 18299402 PMC2275382

[17] Pedranzini L, Mottadelli F, Ronzoni S, Rossella F, Ferracin M, Magnani I, et al Differential cytogenomics and miRNA signature of the Acute Myeloid Leukaemia Kasumi-1 cell line CD34+38- compartment. Leukemia research. 2010;34(10):1287–95. 10.1016/j.leukres.2010.02.012 . 20227111

[18] He Y, Jiang X, Chen J. The role of miR-150 in normal and malignant hematopoiesis. Oncogene. 2014;33(30):3887–93. 10.1038/onc.2013.346 . 23955084

[19] Gerloff D, Grundler R, Wurm AA, Brauer-Hartmann D, Katzerke C, Hartmann JU, et al NF-kappaB/STAT5/miR-155 network targets PU.1 in FLT3-ITD-driven acute myeloid leukemia. Leukemia. 2015;29(3):535–47. 10.1038/leu.2014.231 . 25092144 PMC4490787

[20] Zhang N, Wei X, Xu L. miR-150 promotes the proliferation of lung cancer cells by targeting P53. FEBS letters. 2013;587(15):2346–51. 10.1016/j.febslet.2013.05.059 . 23747308

[21] Wang DT, Ma ZL, Li YL, Wang YQ, Zhao BT, Wei JL, et al miR-150, p53 protein and relevant miRNAs consist of a regulatory network in NSCLC tumorigenesis. Oncology reports. 2013;30(1):492–8. 10.3892/or.2013.2453 . 23670238

[22] Chen Y, Jacamo R, Konopleva M, Garzon R, Croce C, Andreeff M. CXCR4 downregulation of let-7a drives chemoresistance in acute myeloid leukemia. The Journal of clinical investigation. 2013;123(6):2395–407. 10.1172/JCI66553 23676502 PMC3668829

[23] Gregory PA, Bert AG, Paterson EL, Barry SC, Tsykin A, Farshid G, et al The miR-200 family and miR-205 regulate epithelial to mesenchymal transition by targeting ZEB1 and SIP1. Nature cell biology. 2008;10(5):593–601. 10.1038/ncb1722 . 18376396

