# Supplementary material for: Genome-Wide CRISPR-Cas9 Screen Identifies MicroRNAs That Regulate Myeloid Leukemia Cell Growth
Source: PLoS One. 2016 Apr 15;11(4):e0153689. doi: 10.1371/journal.pone.0153689 (PMC4833428; doi:10.1371/journal.pone.0153689)
Supplement: S1 Supporting Methods — (DOCX) [file pone.0153689.s001.docx]

**Supplemental Experimental Procedures**

**CRISPR-Cas9 Library Screen:**

*Infection:*

Genome-scale CRISPR Knock-Out (GeCKO) v2.0 was purchased from Addgene for application in all lentiCRISPRv2 library screens. Version A of this library, containing 4 unique constructs to each miRNA and 3 per protein-coding gene, was used for our screens. The lentiCRISPRv2 library screen was performed as described previously (Shalem et al., 2014). Briefly, the lentiCRISPRv2 library and packaging plasmids pVSVg and psPAX2 were transfected into 293T cells using Trans-IT to produce virus. Virus was subsequently concentrated and titered. Infection was performed at a calculated MOI=0.3, with an expected library coverage of 250x for each individual infection. The lentiCRISPRv2 library was then transduced into MV4-11 cells with 8 ug/ml polybrene via spin infection for 2 hours at 30°C. Two days following spin infection, cells were selected with 2 ug/ml puro for a total of 7 days. Following puro selection, cells were allowed to grow for 16 additional days before completing the experiment. Media was added and the cultured cells were expanded onto additional plates to keep cells at an appropriate culture density. No cells were discarded during the CRISPR-Cas9 screening process in order to prevent loss of constructs with low initial counts, or over-representation of constructs with higher initial counts. Cells were harvested and DNA was extracted 2 days post-infection (TP0), as well as at the end of the experiment (TP23) using the Qiagen DNeasy Blood and Tissue Kit to evaluate library representation at initial and final time points.

*PCR detection of lentiCRISPRv2 constructs:*

PCR on gDNA was performed using Phusion Hot Start II high fidelity DNA polymerase following manufacturer’s instructions (Thermo Scientific). A two-step PCR method, similar to that described ^1^, was employed to amplify inserted sgRNA sequences and append molecular barcodes and Illumina adapter sequences. We modified our method to be compatible with Illumina TruSeq sequencing and obtain sufficient sequence information from a single-end 50-cycle sequencing run. In the first PCR, primers were designed to target the flanking sgRNA sequences and add partial Illumina adapter sequences. A mix of forward primers was used that contained between 2 and 9 variable nucleotides in order to increase the library complexity. Reaction conditions were as follows: 98 **°**C denature for 2 minutes followed by 20 cycles of 98 **°**C for 20 seconds, 62.0 **°**C anneal for 30 seconds, 72 **°**C extension for 20 seconds, and a final single extension step at 72 **°**C for 2 minutes. Multiple reactions per library were performed to ensure adequate input library coverage, then combined and gel extracted and used as a template for the second PCR. The second PCR primers added the rest of the Illumina TruSeq adapter sequences, including the 6-nucleotide molecular barcode added by the reverse primer. The reaction conditions were the same as the first PCR except only 16 cycles were used with an anneal temperature of 63.3 **°**C.

*Analysis:*

DNA sequencing was performed on an Illumina HiSeq 2000 machine at the University of Utah DNA Sequencing Core Facility. The primer sequence was removed from each read using cutadapt 1.6. Reads that contained primer sequence and had a minimum length of 20bp after trimming were aligned using Bowtie-1.0.0 to a reference containing all sgRNAs. Up to three mismatches were allowed, as long as there was only a single alignment location. Read counts for the multiple sgRNAs for each gene were then compiled to obtain gene counts for each miRNA and protein-coding gene in the lentiCRISPRv2 library at initial and final time points. These gene counts were used in DESeq2 in order to determine normalized log2 fold change in growth from initial to final time points and significant p-values (<0.05). Data was compiled from three independent library infections to generate all figures. Protein-coding genes and mature miRNAs determined not to be expressed, or expressed at very low levels, in MV4-11 cells by RNA-seq (FPKM <1 or <10 counts, respectively) were removed from the analysis. miRNAs were classified as conserved based off of “broadly conserved” and “conserved” miRNA families from TargetScan.org website version 6.2. miRNAs predicted to target known oncogenes/TSGs were extracted from “Predicted Conserved Targets” file available on TargetScan.org website. Classifications were determined by looking for exact matches between the CRISPR name and the “mirBase ID” column stripped of the -3p and -5p designators in the “miR Family” file available on the TargetScan.org website. If no match was found and the CRISPR name was suffixed with a hyphen and number, it was removed and checked again for matches. Heat maps were generated using the R package “pheatmap” using “Euclidean” as the distance measure and “complete” as the clustering method.

**CRISPR-Cas9 Lentivector Infections**: Single CRISPR-Cas9 lentivector infections were performed using the general protocol outlined above. Unique sgRNA sequences were cloned into CRISPR-Cas9 lentiCRISPRv2 construct (a gift from Feng Zhang; Addgene plasmid #52961) containing either a puro resistance or GFP selection marker. sgRNA sequences for each individual vector are listed below:

STAT5A-CR1= GGTGAACGGCCATGGCGGGC (puro)

STAT5A-CR2= GCAAGTAGTGCCGGACCTCGA (puro)

Ago2-CR1= TAACGCCTGCAAGCTCACGC (GFP)

Drosha-CR1= CGTCCTATGACCAACAATGC (GFP)

miR-150-CR1= CCAGGGTCTGAGCCCAGCAC (GFP)

p53-CR1= CCCCGGACGATATTGAACAA (GFP)

miR-155-CR1= GTTAATGCTAATCGTGATAG (GFP)

miR-155-CR2= GCTAATATGTAGGAGTCAGT (GFP)

**Expression profiling:** Total RNA was isolated using the miRNeasy spin column kit (Qiagen). Expression of small RNAs and long RNAs in MV4-11 cells was assessed via RNA-seq. NEB library prep (for small RNA reads) and RiboZero treatment/library preparation (for long RNAs) were performed at the University of Utah DNA Sequencing Core Facility, followed by stranded RNA sequencing using Illumina HiSeq 2000 sequencing. Aligned reads (miRBase) were used in DESeq2, which normalizes the signal and determines differential expression. Counts to mature miRNAs and the primary transcripts (miRBase v21 annotations) were generated using the small RNA data. Mature miRNAs with at least 10 counts across the three replicates were considered expressed.
